
JOURNAL INFORMATION
==============================
NLM Title Abbreviation: Biospektrum (Heidelb)
Journal ID: Biospektrum (Heidelb)
NLM Journal ID: 9615685

Biospektrum

ISSN: 0947-0867
EISSN: 1868-6249

ARTICLE INFORMATION
==============================
PMCID: PMC8853259
PMID: 35194332
DOI: 10.1007/s12268-022-1712-y
Article ID: 1712
Article version: 1
Subjects: Wissenschaft Special

Wie sich COVID-19 in der 3D-Zellkultur simulieren lässt

Wilflingseder Doris doris.wilflingseder@i-med.ac.at

Posch Wilfried
grid.5361.1 0000 0000 8853 2677 Institut für Hygiene und Medizinische Mikrobiologie, Medizinische Universität Innsbruck, Schöpfstraße 41/R311, A-6020 Innsbruck, Österreich
Electronic publication date: 2022 Feb 13
Print publication date: 2022
Volume: 28
Issue: 1
First page: 43
Last page: 46
Copyright: © Die Autorinnen und Autoren 2022
License: Dieser Artikel wird unter der Creative Commons Namensnennung 4.0 International Lizenz veröffentlicht, welche die Nutzung, Vervielfältigung, Bearbeitung, Verbreitung und Wiedergabe in jeglichem Medium und Format erlaubt, sofern Sie den/die ursprünglichen Autor(en) und die Quelle ordnungsgemäß nennen, einen Link zur Creative Commons Lizenz beifügen und angeben, ob Änderungen vorgenommen wurden. Die in diesem Artikel enthaltenen Bilder und sonstiges Drittmaterial unterliegen ebenfalls der genannten Creative Commons Lizenz, sofern sich aus der Abbildungslegende nichts anderes ergibt. Sofern das betreffende Material nicht unter der genannten Creative Commons Lizenz steht und die betreffende Handlung nicht nach gesetzlichen Vorschriften erlaubt ist, ist für die oben aufgeführten Weiterverwendungen des Materials die Einwilligung des jeweiligen Rechteinhabers einzuholen. Weitere Details zur Lizenz entnehmen Sie bitte der Lizenzinformation auf http://creativecommons.org/licenses/by/4.0/deed.de.
License URL: https://creativecommons.org/licenses/by/4.0/

issue-copyright-statement: © The Author(s), under exclusive licence to Springer-Verlag GmbH Deutschland, ein Teil von Springer Nature 2022

==============================
Excessive inflammation triggered by a hitherto undescribed mechanism is a hallmark of severe SARS-CoV-2 infection and is associated with enhanced pathogenicity and mortality. Complement hyper activation promotes lung injury and was observed in patients suffering from MERS-CoV, SARS-CoV-1 and SARS-CoV-2 infections. To evaluate the very first interactions of SARS-CoV-2 patient isolates with human epithelial tissues, 3D models of the human respiratory tract as well as lung organoids are highly suitable.

▲ **Abb. 1:** Untersuchung von COVID-19 im standardisierten 3D-Zellkulturmodell. Das Barrieremodell des respiratorischen Trakts besteht aus hoch differenziertem Flimmerepithel (braun) sowie mukusproduzierenden Zellen (blau, violett). Das pseudostratifizierte Luft-Flüssig-Interphase-Modell kann durch Stammzellen im System (türkis) erneuert werden, sodass das Zellkulturmodell sich auch für Langzeitversuche eignet – es kann bis zu 2 Jahren in Kultur gehalten werden. Die Epithelien können nach Differenzierung von der apikalen (Luft-)Seite mit SARS-CoV-2 infiziert werden, um die Situation im Körper möglichst naturgetreu nachzustellen. Des Weiteren können die Gewebe mit antiviralen Sprays von der apikalen Seite her behandelt werden und deren Wirkung gegen SARS-CoV-2 getestet werden. Immunzellen von Interesse (hier dendritische Zellen) können bei Bedarf leicht in das 3D-Zellkultursystem integriert und so auch zusätzliche Faktoren, die im Körper eine Rolle während COVID-19 spielen, analysiert werden. Erstellt mit BioRender.

■ Frühe Ereignisse, die direkt nach der Übertragung von SARS-CoV-2 auf Gewebe des Atmungstrakts auftreten, bestimmen den Verlauf der Erkrankung maßgeblich. Bei einigen Patienten führt eine Infektion mit COVID-19 zu einer überschießenden Immunantwort und damit verbunden der Erzeugung eines proinflammatorischen Milieus. Die Entwicklung eines Zytokinsturms und eine akute Lungenschädigung (ARDS, *acute respiratory distress syndrome*) sind unerwünschte Folgen von COVID-19 und führen zu einer erhöhten Morbidität und Mortalität durch Gewebsverletzungen sowie Multiorganversagen [1–6].

Mit neuartigen 3D-Zellkultursystemen lassen sich Wechselwirkungen zwischen Krankheitserregern und immunkompetenten Barrieremodellen simulieren und studieren, was auch Angriffsflächen für neue Therapieoptionen möglich macht (**Abb. 1**). Daher spielt auf der Suche nach neuen Medikamenten und Therapien auch die Grundlagenforschung eine wesentliche Rolle und besonders innovative, vielversprechende und tierversuchsfreie Ansätze können das Verständnis von ersten SARS-CoV-2-Interaktionen an den Atemwegsepithelien verbessern.

Das gesamte respiratorische Epithel von der Nasenhöhle bis hinab zu den Alveolen ist konstant Krankheitserregern ausgesetzt. Wenn die Krankheitserreger in der Lage sind, das mukusproduzierende, pseudostratifizierte Flimmerepithel zu durchbrechen, muss spontan die angeborene Immunantwort aktiviert werden. In diesem Kontext wurde gezeigt, dass die Flimmerepithelien, die eigentlich nicht zu unserem Immunsystem gehören, Proteine des Komplementsystems produzieren und sezernieren können – eine sofortige Immunreaktion unseres Körpers [7]. Auch in schwer erkrankten COVID-19-Patienten wurde beschrieben, dass Komplement bei der Verstärkung der Entzündungsreaktion eine wichtige Rolle spielen könnte. Das Komplementsystem, das im Normalfall vor Virusinfektionen schützen soll, könnte somit während schwerer COVID-19-Verläufe fatal wirken und die Gewebszerstörung vor-

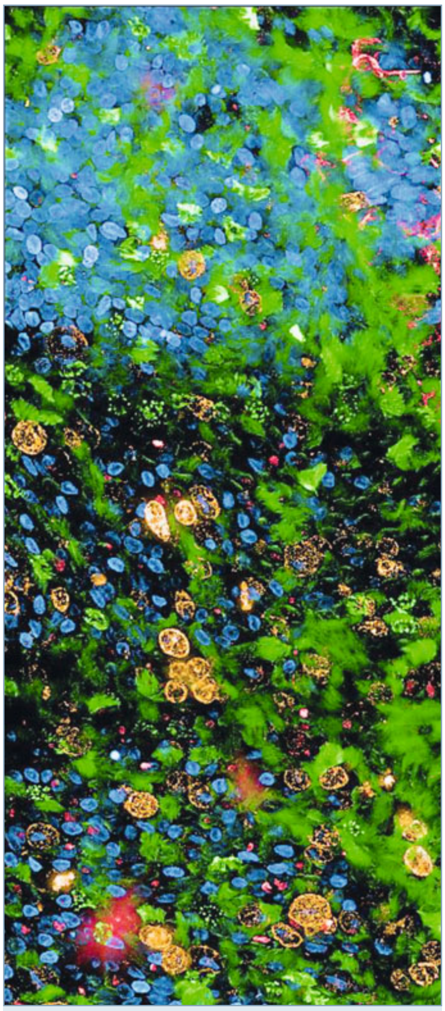

▲ **Abb. 2:** SARS-CoV-2 zerstört die Gewebeintegrität und verursacht Mukushypersekretion im 3D-Barrieremodell. Nach 2 Tagen Infektion mit einer hohen Viruslast eines SARS-CoV-2-Patientenisolats (rot) sieht man bereits eine massive Zerstörung des Gewebeverbands, erkennbar an fragmentierten Zellkernen (Nuklei, blau) und Löchern in den Geweben. Des Weiteren sieht man eine Mukusüberproduktion (orange) in infizierten Epithelien. Es werden sowohl Flimmerepithelzellen (grün) als auch mukusproduzierende Zellen (orange) durch das SARS-CoV-2-Wildtyp-Virus, welches zu Beginn der Pandemie isoliert wurde, infiziert.

antreiben, wenn das Flimmerepithel durch lokale Komplement- und damit verbunden Anaphylatoxinproduktion die Entzündungsreaktion verschlimmert.

Durch Simulierung einer akuten SARS-CoV-2-Infektion in einem über die letzten Jahre optimierten und standardisierten Luft-Flüssig-Modell des oberen und unteren respiratorischen Trakts [8, 9] sowie in Lungenorganoiden, kann quasi im Livemodus beobachtet werden, wie Epithelzellen auf die Infektion reagieren, welche Zytokine und Anaphylatoxine ausgeschüttet werden, wie schnell Viruspartikel weitertransportiert werden, beziehungsweise welche Gewebszerstörung das Virus verursacht. Dies sind nur einige der vielen Analysen, die in der 3D-Zellkultur durchgeführt werden können. Interessant ist auch die Behandlung solcher Kulturen mit antiviralen oder nicht konventionellen, aber vielversprechenden, Medikamenten, wie z. B. Komplementblockern. Unter Verwendung der 3D-Zellkulturmodelle wurden folgende Fragestellungen im Jahr 2020 sofort nach Auftreten von SARS-CoV-2 in Europa und Expansion sowie Charakterisierung von Virusisolaten aus Patienten bearbeitet.

## Was passiert direkt nach SARS-CoV-2-Übertragung an den epithelialen Barrieren?

Hoch differenzierte, schleimproduzierende Flimmerepithelien des oberen und unteren Respirationstrakts wurden mit SARS-CoV-2-Patientenisolaten infiziert und nach zwei Tagen mittels Immunfluoreszenz analysiert. Die Gewebemodelle zeigten eine starke Infektion mit SARS-CoV-2 unabhängig vom Zelltyp – sowohl Wimper- als auch mukusproduzierende Zellen wurden von SARS-CoV-2 infiziert (**Abb. 2**, [10]). Die Gewebe wurden signifikant zerstört, wie man anhand von fragmentierten Zellkernen erkennen konnte [10]. Des Weiteren fiel in den infizierten Gewebemodellen sofort eine Hypersekretion von Schleim sowie Schleimpfropfenbildung auf. Im Gegensatz dazu waren in nicht infizierten, scheinbehandelten Kontrollen, Mukuspfropfen nicht nachweisbar [10]. Bei der Analyse schwerkranker COVID-19-Patienten mit Atemwegsobstruktion und Atemversagen wurden sowohl Mukuspfropfenbildung als auch -hypersekretion in Kombination mit einer Hyperaktivierung proinflammatorischer Zytokine beschrieben [11]. Diese Veränderungen zusammen mit einer reduzierten mukoziliären Clearance, die ebenfalls in unseren infizierten 3D-Modellen nachgewiesen wurde, bilden einen Teufelskreis, der zur – während COVID-19 beobachteten – Gewebszerstörung führt.

## Welche Mechanismen des angeborenen Immunsystems werden nach SARS-CoV-2-Infektion von humanen respiratorischen Geweben aktiviert?

In einem nächsten Schritt wurden die immunologischen Antworten auf die SARS-CoV-2-Infektion, die von den – nicht zum Immunsystem gehörenden – Epithelzellen produziert und sezerniert wurden, untersucht [12]. Da Komplementhyperaktivierung Lungenschädigung verursacht und nicht nur in Patienten, die an MERS-CoV und SARS-CoV-1 erkrankt waren, sondern auch bei SARS-CoV-2-infizierten Individuen festgestellt wurde, studierten wir lokale Komplementproduktion in 3D-Zellkulturmodellen, die mit SARS-CoV-2 infiziert wurden [12]. Nach Infektion wurden Viruslast, Infektiosität der neu produzierten Viruspartikel, intrazelluläre Komplementaktivierung, Entzündungsmechanismen und Gewebszerstörung mittels quantitativer RT-PCR, Hochdurchsatzscreening-Analysen, Plaque-Assays, Luminex-Analysen und Erfassung des transepithelialen Widerstands gemessen [12]. Quantitative Analysen all dieser Parameter sind in **Abbildung 3** dargestellt. Im SARS-CoV-2-infizierten 3D-Zellkulturmodell konnte bei starker Infektion eine vollständige Zerstörung der epithelialen Integrität beobachtet werden, die mit einer verstärkten Produktion der Komplementkomponente 3 (C3), einer Bildung des Membranangriffskomplexes (MAC) sowie einer Ablösung von Zellen aus dem Gewebeverband und einer Freisetzung des Virus in die Überstände verbunden war. SARS-CoV-2-Infektion wurde nicht nur an der oberen Epithelschicht nachgewiesen, sondern die Viruspartikel drangen tief in das pseudostratifizierte Epithel ein. Dies ging einher mit einer C3-Aktivierung und auch C3-Ablagerung auf toten oder beschädigten Zellen. Neben einer signifikant höheren C3-Aktivierung in SARS-CoV-2-infizierten Zellen des Respirationstrakts wurden auch signifikant höhere Konzentrationen des Anaphylatoxins C3a desArg sowie den proinflammatorischen Zytokinen Interleukin(IL)-6, Monozyten-chemotaktisches Protein-1 (MCP-1), IL-1$\alpha$ und RANTES nachgewiesen [12]. Die Komplementaktivierung zu stoppen war somit ein logisches, nächstes Vorgehen, um die SARS-CoV-2-induzierten Entzündungsreaktionen und die Gewebszerstörung zu blockieren. Daher wurden Antagonisten der Anaphylatoxinrezeptoren C3aR und C5aR verwendet und deren Effekt mit oben genannten Methoden analysiert. Die Rezeptoren wurden sowohl prophylaktisch als auch einige Stunden nach erfolgter SARS-CoV-2-Infektion zugegeben. Unabhängig vom Zugabemodus konnte gesehen werden, dass die Blockierung von C3aR die virale

Infektion und die Entzündungsreaktion in den Atemwegsgeweben verringerte. Einen größeren Effekt hatte jedoch der C5aR-Antagonismus – Blockierung dieses Rezeptors auf der basolateralen, also der Körperinnenseite zugewandten, Seite stellte die Gewebsintegrität vollständig wieder her, regulierte die lokale Komplement-C3-Produktion signifikant herunter und brachte damit verbunden die C3a- und proinflammatorischen Spiegel wieder auf den Normalwert zurück. Es wurde auch gezeigt, dass C5aR-Antagonismus mit niedriger Viruslast korreliert [12].

Unsere Ergebnisse weisen auf eine neuartige therapeutische Interventionsstrategie hin, bei der Hochrisikopatienten in einem frühen Stadium des Krankheitsverlaufs Komplementinhibitoren verabreicht werden, um eine übermäßige Entzündungsreaktion und die damit verbundenen pathologischen Folgen zu verhindern. Verschiedene Komplementinhibitoren befinden sich derzeit in fortgeschrittenen Stadien klinischer SARS-CoV-2-Studien (z. B. AMY-101, Eculizumab, Ravulizumab) und könnten über Vernebler in die Lunge verabreicht werden [13].

### Wie kann der Eintritt von SARS-CoV-2 ins Nasen-/Bronchialepithel verhindert werden?

Um die Virusübertragung und die damit verbundene Gewebszerstörung und Komplementhyperaktivierung zu verhindern, sind sofortige Interventionen nötig – daher wurde im 3D-Zellkulturmodell der Nase und des oberen Respirationstrakts ein antivirales Mundspray (ColdZyme/ViruProtect) ausgetestet [14]. Die Anwendung von ColdZyme auf vollständig differenziertes, polarisiertes menschliches Epithel, das an einer Luft-Flüssigkeits-Interphase (ALI) kultiviert wurde, blockierte die Bindung von SARS-CoV-2 vollständig wie auch die lokale Komplement-C3-Produktion und die damit verbundene Infektion und Zerstörung des Gewebemodells.

Wie bereits oben beschrieben, verursachte die SARS-CoV-2-Infektion ohne vorherige Behandlung mit ColdZyme eine Komplementhyperaktivierung einhergehend mit einer überschießenden Entzündungsreaktion und hoher Virusproduktion. Durch einmalige Vorbehandlung der Gewebe mit ColdZyme-Mundspray wurden diese Effekte vermieden und so der Transfer von SARS-CoV-2 verhindert [14]. Unsere *in vitro*-Daten deuten darauf hin, dass ColdZyme-Mundspray einen Einfluss auf die Prävention von COVID-19 haben kann und dass es wichtig ist, bereits zugelassene, antivirale Medikamente auf ihre Wirksamkeit gegen SARS-CoV-2 zu überprüfen.

Humane 3D-Zellkultursysteme eignen sich – wie wir zeigen konnten – nicht nur für die Untersuchung von ersten Wirt-Pathogen-Interaktionen und Virusdynamiken, sondern bieten auch einen Mehrwert vor allem in der präklinischen Phase für die Testung der Wirkung neuer und innovativer therapeutischer oder umfunktionierter (*repurposed drugs*) Interventionen. ■

Hier steht eine Anzeige.

Springer

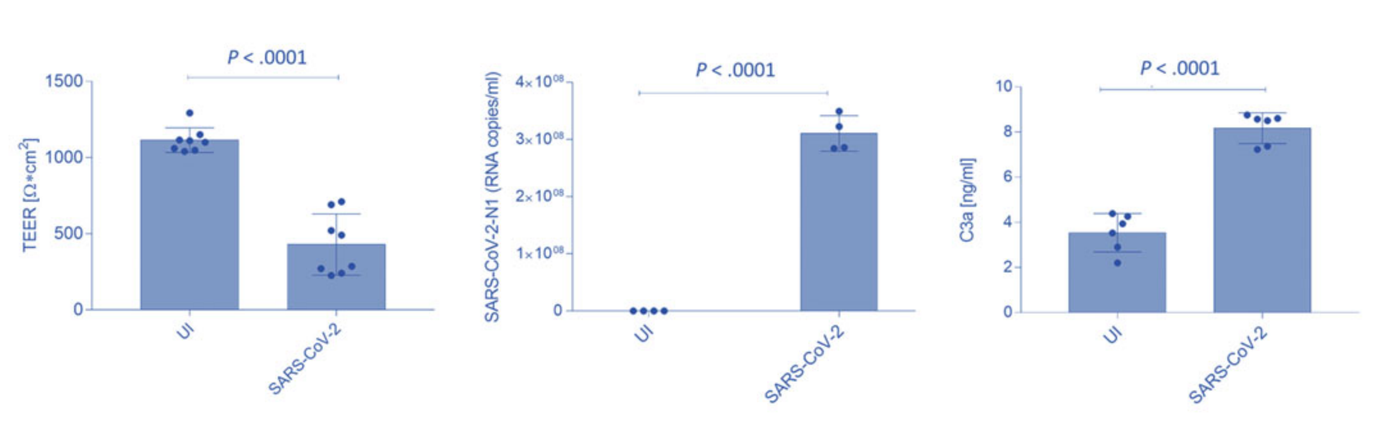

▲ **Abb. 3:** Quantitative Analysen der Gewebeintegrität, der Viruslast und Anaphylatoxinproduktion. Links: Nach 3 Tagen Infektion mit einem SARS-CoV-2-Patientenisolat sieht man bereits eine massive Zerstörung des Gewebeverbands, wie durch Messung des transepithelialen elektrischen Widerstandes (TEER, *transepithelial electrical resistance*) detektiert wurde. Ein hoch signifikanter Abfall (P< 0,0001) der TEER-Werte ist im SARS-CoV-2-infizierten verglichen mit nicht infiziertem Kontrollgewebe (UI) erkennbar. Mitte: SARS-CoV-2-Virus wurde in hoher Konzentration an Tag 3 nach Infektion von ALI-Zellkulturmodellen im basolateralen Überstand mittels RT-PCR und absoluter Quantifizierung analysiert, während in nicht infizierten Kontrollgeweben – wie erwartet – kein Virus nachweisbar war. Rechts: Signifikant höhere Freisetzung (P<0,0001) des Anaphylatoxins C3a wurde in SARS-CoV-2-infizierten versus nicht infizierten Geweben detektiert. Signifikante Unterschiede wurden mittels unpaired Student's t-Test in der GraphPad Prism Software ermittelt. Abbildung aus [12], CC BY 4.0.


Funding note

Open access funding provided by University of Innsbruck and Medical University of Innsbruck.

Doris Wilflingseder Biologiestudium und Promotion. 2008–2009 Forschungsarbeit am University College London, UK. 2005–2012 Arbeitsgruppenleiterin am Institut für Hygiene und Medizinische Mikrobiologie, Medizinische Universität Innsbruck. 2012–2019 Assoziierte Professorin und Arbeitsgruppenleiterin am Institut für Hygiene und Medizinische Mikrobiologie, Medizinische Universität Innsbruck. Seit 2020 Universitätsprofessorin für Infektionsbiologie, Medizinische Universität Innsbruck.

Wilfried Posch Molekularbiologiestudium an der Medizinischen Universität Innsbruck. 2009 Promotion. 2009 University College London, UK. 2012 Postdoc am INSERM, Paris, Frankreich. 2010–2021 Arbeitsgruppenleiter am Institut für Hygiene und Medizinische Mikrobiologie der Medizinischen Universität Innsbruck. Seit 2021 Assistenzprofessor für Emerging Infectious Diseases, Medizinische Universität Innsbruck.


Literatur

[1] Magro G COVID-19: review on latest available drugs and therapies against SARS-CoV-2. Coagulation and inflammation cross-talking Virus Res 2020 286 198070 10.1016/j.virusres.2020.198070 32569708 PMC7305708
[2] Zhu N Zhang D Wang W A novel coronavirus from patients with pneumonia in China, 2019 N Engl J Med 2020 382 727 733 10.1056/NEJMoa2001017 31978945 PMC7092803
[3] Tang N Li D Wang X Sun Z Abnormal coagulation parameters are associated with poor prognosis in patients with novel coronavirus pneumonia J Thromb Haemost 2020 18 844 847 10.1111/jth.14768 32073213 PMC7166509
[4] Wang D Hu B Hu C Clinical characteristics of 138 hospitalized patients with 2019 novel coronavirus-infected pneumonia in Wuhan, China JAMA 2020 323 1061 1069 10.1001/jama.2020.1585 32031570 PMC7042881
[5] Chen G Wu D Guo W Clinical and immunologic features in severe and moderate coronavirus disease 2019 J Clin Invest 2020 130 2620 2629 10.1172/JCI137244 32217835 PMC7190990
[6] Huang C Wang Y Li X Clinical features of patients infected with 2019 novel coronavirus in Wuhan, China Lancet 2020 395 497 506 10.1016/S0140-6736(20)30183-5 31986264 PMC7159299
[7] Gu H Fisher AJ Mickler EA Contribution of the anaphylatoxin receptors, C3aR and C5aR, to the pathogenesis of pulmonary fibrosis FASEB J 2016 30 2336 2350 10.1096/fj.201500044 26956419 PMC4871799
[8] Zaderer V Hermann M Lass-Florl C Turning the world upside-down in cellulose for improved culturing and imaging of respiratory challenges within a human 3D model Cells 2019 8 1292 10.3390/cells8101292 PMC6830077 31640299
[9] Chandorkar P Posch W Zaderer V Fasttrack development of an in vitro 3D lung/immune cell model to study Aspergillus infections Sci Rep 2017 7 11644 10.1038/s41598-017-11271-4 28912507 PMC5599647
[10] Posch W Lass-Flörl C Wilflingseder D SARS-CoV-2-infected primary human airway epithelia illustrate mucus hypersecretion J Allergy Clin Immunol 2021 148 909 10.1016/j.jaci.2021.05.047 34284045 PMC8285928
[11] Khan MA Khan ZA Charles M Cytokine storm and mucus hypersecretion in COVID-19: review of mechanisms J Inflamm Res 2021 14 175 189 10.2147/JIR.S271292 33519225 PMC7838037
[12] Posch W Vosper J Noureen A C5aR inhibition of nonimmune cells suppresses inflammation and maintains epithelial integrity in SARS-CoV-2-infected primary human airway epithelia J Allergy Clin Immunol 2021 147 2083 2097.e6 10.1016/j.jaci.2021.03.038 33852936 PMC8056780
[13] Jodele S Köhl J Tackling COVID-19 infection through complement-targeted immunotherapy Br J Pharmacol 2021 178 2832 2848 10.1111/bph.15187 32643798 PMC7361469
[14] Posch W Vosper J Zaderer V ColdZyme maintains integrity in SARS-CoV-2-infected airway epithelia mBio 2021 12 e00904 21 10.1128/mBio.00904-21 33906927 PMC8092264
